# Visceral Adipose Tissue from Obese and Diabetic Mice Induces Tau Pathogenesis

**DOI:** 10.1002/alz70855_105597

**Published:** 2025-12-24

**Authors:** Miriam Bettinetti‐Luque, Laura Trujillo‐Estrada, Juana Andreo‐Lopez, Cynthia Campos‐Moreno, Mario Morales‐Cabello, Ian Perea‐Santaella, Elisabeth Sanchez‐Mejias, Celia Da Cunha, Frank M LaFerla, Raquel Sanchez‐Varo, Antonia Gutierrez, David Baglietto‐Vargas

**Affiliations:** ^1^ University of Malaga/CIBERNED/IBIMA, Málaga, Spain; ^2^ University of California, Irvine, Irvine, CA, USA

## Abstract

**Background:**

Alzheimer's disease (AD) is a complex disorder involving multiple cellular and molecular mechanisms. Recent evidence suggests that metabolic alterations play a crucial role in AD progression. Likewise, diabetes and obesity—two major metabolic diseases—are well‐established risk factors for AD. These conditions are associated with a significant expansion of visceral adipose tissue. Here, we hypothesize that visceral adipose tissue may act as a key mediator between peripheral metabolic dysfunction and brain illnesses.

**Method:**

We used histological stains, immunohistochemistry, and biochemical assays to analyze changes in visceral adipose tissue from WT, db/db, and WT mice subjected to a high‐fat diet (HFD). Additionally, we performed adipose tissue transplants from db/db donors into 3xTg‐AD mice and from HFD‐fed WT donors into Tau P301S mice to evaluate potential alterations in tau pathology in these distinct AD models.

**Result:**

Our study shows that 3xTg‐AD mice receiving fat pads from db/db donors exhibited a significant increase in tau pathology, driven by elevated p25/p35 expression, compared to 3xTg‐AD mice receiving fat from standard‐diet WT mice. This tau accumulation was associated with increased IL‐1β levels and robust microglial activation. Similarly, Tau P301S mice transplanted with fat from HFD‐fed WT donors also displayed exacerbated tau pathology and neuroinflammation, indicating that metabolic dysfunction induced major changes in neuroinflammation that contributed to tau pathology.

**Conclusion:**

Overall, our study highlights a novel interplay between AD and metabolic disorders, demonstrating that visceral adipose tissue from both genetically obese/diabetic and diet‐induced obese mice exacerbates tau pathology via immune system activation. These findings reinforce the role of metabolic dysfunction in AD and suggest visceral adipose tissue as a potential target for therapeutic intervention.

